# Tracing Soil CO_2_ Fluxes under Drying-Rewetting Cycles: Isotopic Insights from an Automatic Soil Incubation System

**DOI:** 10.1021/acs.est.5c10776

**Published:** 2026-03-06

**Authors:** Yuedan Zhao, Nan Lu, Susan Trumbore, Martin Goebel, Karl Kuebler, Hui Wang, Marion Schrumpf, Kai Wang, Cong Wang, Bojie Fu, Jianbei Huang

**Affiliations:** † 28300 Max Planck Institute for Biogeochemistry, Hans-Knöll-Straße 10, Jena 07745, Germany; ‡ State Key Laboratory of Regional and Urban Ecology, Research Center for Eco-Environmental Sciences, 12381 Chinese Academy of Sciences, Beijing 100085, China; § University of Chinese Academy of Sciences, Beijing 100049, China

**Keywords:** drying-rewetting cycles, soil CO_2_ pulses and emissions, stable isotope and radiocarbon, drylands

## Abstract

Climate change is expected to increase the intensity and frequency of droughts and heavy rainfall events globally, with significant consequences on the terrestrial carbon cycle. One of the most critical yet highly variable components of the carbon cycle is the soil CO_2_ pulse triggered by precipitation events in dryland ecosystems. To examine the processes underlying the soil CO_2_ pulse under changing precipitation patterns, we developed a unique Online Automatic Soil Incubation System (OASIS) that allows (1) accurate manipulation of drying and rewetting regimes; (2) continuous monitoring of soil CO_2_ fluxes; and (3) identification of their isotopic sources (^13^C and ^14^C). Using OASIS, we investigated how normal and extreme drying-rewetting cycles (NDWC vs EDWC) influence CO_2_ pulse emissions and their isotopic signatures from soils of the Loess Plateau, while controlling for total water input. Our results showed that EDWC induced a rapid peak in the CO_2_ release rate within minutes, but this was offset by reduced emissions during the dry phase compared to NDWC. In addition, total CO_2_ release was strongly influenced by CO_2_ influx through dissolution, which was limited during the prolonged dry phase under EDWC. Isotopic data indicated that the CO_2_ pulse that originated from substrates was derived from recent plant carbon input within minutes of rewetting and was potentially influenced by exchange with the inorganic carbon pool, followed by contributions from bulk SOC that may have persisted for hundreds to thousands of years. These findings underscore the importance of accounting for CO_2_ pulses driven by substrate availability and different carbon sources, which is crucial for improving model predictions of soil CO_2_ flux and carbon storage in drylands under changing precipitation patterns. We highlight the applications of OASIS in revealing how interacting climatic, biological, and physicochemical factors drive soil greenhouse gas emissions.

## Graphical abstract

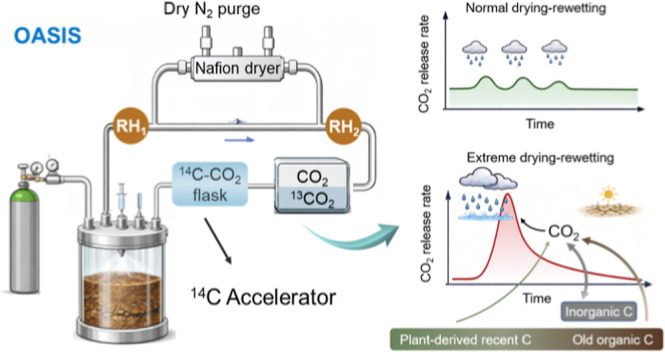

## Introduction

Climate change poses a threat to terrestrial ecosystem functioning by altering the frequency and intensity of extreme events like dry spells and heavy precipitation.^1^ These extreme events can have profound impacts on dryland ecosystems, which are defined by an aridity index (the ratio of annual precipitation to potential evapotranspiration) of less than 0.65.^2^ These ecosystems cover more than 45% of the Earth’s land area and are continuing to expand.^2^ Dryland ecosystems have been shown to dominate the interannual variability and long-term trends of the terrestrial carbon sink due to their high sensitivity to precipitation changes.^3^ A distinctive feature of carbon fluxes in drylands is the pulse release of CO_2_ from soils following an increase in soil moisture caused by precipitation, also known as the “Birch effect”.^4^ For example, Metz et al.^5^ showed that the interannual variability of Australia’s CO_2_ balance is largely driven by the soil CO_2_ pulse that occurs shortly after the onset of rainfall in dryland regions. However, assessing and modeling such ecosystem and soil carbon fluxes in drylands remain largely uncertain,^6,7^ due to limited mechanistic understanding of how changing precipitation patterns, particularly precipitation intensity and frequency, which may influence soil CO_2_ fluxes and the underlying processes.^7^

Investigations on the soil CO_2_ pulse are often conducted in incubation chamber experiments. Many studies have shown that rewetting after severe drying can lead to larger soil CO_2_ pulses.^8,9^ However, there is no consensus on how drying-rewetting cycles influence the overall emission rates and cumulative emissions of CO_2_. This uncertainty arises partly because the observed responses may depend on multiple experimental factors, including the time of sampling,^10^ the moisture level of the control treatment,^11^ and the intensity and duration of the drying-rewetting treatment. Lacking continuous measurements of soil CO_2_ emissions, it is difficult to capture the fast responses and temporal dynamics of soil CO_2_ emissions to changing water conditions.^10^ There are also several limitations related to experimental treatments and their setup for assessing CO_2_ pulse studies: (1) constant soil moisture conditions are unrealistic,^9^ especially in drylands where soil water conditions have large fluctuations; (2) desiccant dryers (typically silica gel) are commonly used to dry the air in the headspace of the chamber, lacking precise control over the duration and intensity of the drying treatment;^7^ (3) the manipulation of multiple drying-rewetting cycles is often achieved by adding more water compared with the constant moisture treatment, thus confounding interpretations of how changes in precipitation regimes alone—without changes in the amount of total precipitation—may alter soil CO_2_ pulses. Addressing these limitations requires novel experimental approaches that combine continuous measurements of soil CO_2_ fluxes with accurate manipulation of drying-rewetting regimes while controlling for differences in water input across treatments.

Most studies on the mechanisms underlying soil CO_2_ pulses have focused on whether these pulses are driven by substrates released through microbial cell lysis and osmolyte breakdown, the disruption of soil aggregates, or the desorption of organic compounds from mineral surfaces.^8,12^ However, the origin of these substrates remains largely uncertain. Radiocarbon is a powerful tool for examining the contributions of young organic carbon (i.e., “bomb” radiocarbon produced by atmospheric thermonuclear weapon testing in the 1960s) and older organic carbon (i.e., carbon subject to long-term radioactive decay of ^14^C).^13^ For example, in one of the few studies that measured ^14^C of soil CO_2_ pulse during drying-rewetting, Schimel et al.^14^ reported a substantial contribution of older carbon (prior to 1960s) to respired CO_2_ after drying-rewetting. In addition to ^14^C, ^13^C is also a valuable tracer for capturing rapid changes in carbon sources during drying-rewetting. For example, the plant-derived young and labile carbon pool has a lower δ^13^C compared to the older and more stable soil organic carbon (SOC) pool that has experienced more microbial processing and isotopic discrimination.^15,16^ Thus, application of ^14^C and ^13^C offers valuable insights into the origin of the substrates fueling the soil CO_2_ pulse during drying-rewetting.^7^

Despite increasing recognition of soil CO_2_ pulses driven by biotic processes, the role of abiotic contributions to soil CO_2_ exchange via soil inorganic carbon (SIC) remains poorly constrained.^17,18^ Emerging evidence shows that dryland soils, especially alkaline soils with high calcium content like those on the Loess Plateau, can abiotically absorb atmospheric CO_2_.^19^ This process is driven by chemical equilibrium where CO_2_ dissolves in soil water and reacts to form bicarbonate and carbonate ions, which subsequently precipitate with calcium or magnesium to form solid carbonates.^20,21^ Measurements of ^14^C and ^13^C can also provide insights into the contribution of such abiotic processes to CO_2_ fluxes, given that CO_2_ derived from SIC tends to be more ^13^C-enriched and more ^14^C-depleted than CO_2_ derived from SOC.^21^

Here, we present a state-of-the-art online automatic soil incubation system (OASIS). The OASIS allows: (1) manipulating soil water balance, i.e., modulating the intensity and duration of soil water input and water loss, thereby creating diverse drying-rewetting scenarios to simulate shifts in precipitation regimes; (2) continuously monitoring soil CO_2_ flux and soil moisture content, to capture rapid changes in soil CO_2_ flux with changing moisture availability; and (3) analyzing gas samples for ^14^C and ^13^C analysis by accelerator mass spectrometry (AMS) and cavity ring-down spectrometer (CRDS), respectively, to identify the origins of soil CO_2_ flux. We present an example of using OASIS to address how changes in precipitation patterns (normal vs extreme drying-rewetting cycles) can alter the magnitude of soil CO_2_ pulses and cumulative emissions and their sources of origin, i.e., old vs young carbon in the soil. We hypothesize that, relative to normal drying and rewetting conditions, (1) extreme drying and rewetting induces larger initial CO_2_ pulses, driven by the greater accumulation of labile substrates during the prolonged dry phase,^7,8^ which can be offset by lower emissions during the subsequent extreme drying phase; (2) extreme drying-rewetting cycles can stimulate microbial decomposition of old organic carbon in soils due to the disruption of soil aggregates or the desorption of organic compounds from mineral surfaces, particularly after multiple cycles. In addition, we incubated sterilized soils to quantify the CO_2_ exchange driven by abiotic processes, thereby partitioning total soil CO_2_ fluxes into biotic and abiotic components.

## Material and Method

### The Modular Design of OASIS

The OASIS consists of three modules: incubation, drying, and measurement (Figures 1 and S1). Briefly, in the incubation module, soils were placed in four independent soil chambers (approximately 0.8 L) made of stainless steel, with gas supplied from a gas tank and passed through a filter and pressure regulator. The pressure, temperature, and relative humidity of the chambers were continuously monitored. In the drying module, the sample gas from the soil chamber passes through a 3.66 m Nafion membrane dryer tube with an inner diameter of 2.18 mm and an outer diameter of 2.74 mm (PPMD-110–144 F, Gasmet Technologies GmbH, Karlsruhe, Germany). The Nafion drier is a copolymer of tetrafluoroethylene (Teflon) and perfluoro-3,6-dioxa-4-methyl-7-octene-sulfonic acid, which efficiently removes water from the sample gas stream to the dry N_2_ purge gas stream based on differences in water vapor pressure, while retaining other analytes like CO_2_, oxides, sulfur compounds, etc.^22^ The relative humidity and temperature of the sample gas stream were measured before and after Nafion drying to calculate water loss and estimate changes in the soil water content. In the measurement module, the sample gas passed through a filter and a CO_2_ sensor (GMP252, Vaisala, Finland; precision ±0.1% CO_2_) for continuous measurements of CO_2_ concentrations, with a 2.3 L flask and a 12 mL exetainer for discrete samplings of ^14^C and ^13^C of CO_2_, respectively. The entire system—including all three modules—is replicated four times, resulting in four fully independent and parallel systems, except for using the same gas supply with individually controlled valves. For one of the four soil incubation chambers, we connected a Picarro (2131-i, Picarro Inc. Santa Clara, CA) to the sampling line for the continuous analysis of ^13^C–CO_2_. All sensors and instruments were connected to a Campbell data logger (CR1000, Campbell, U.K.).

**Figure 1:**
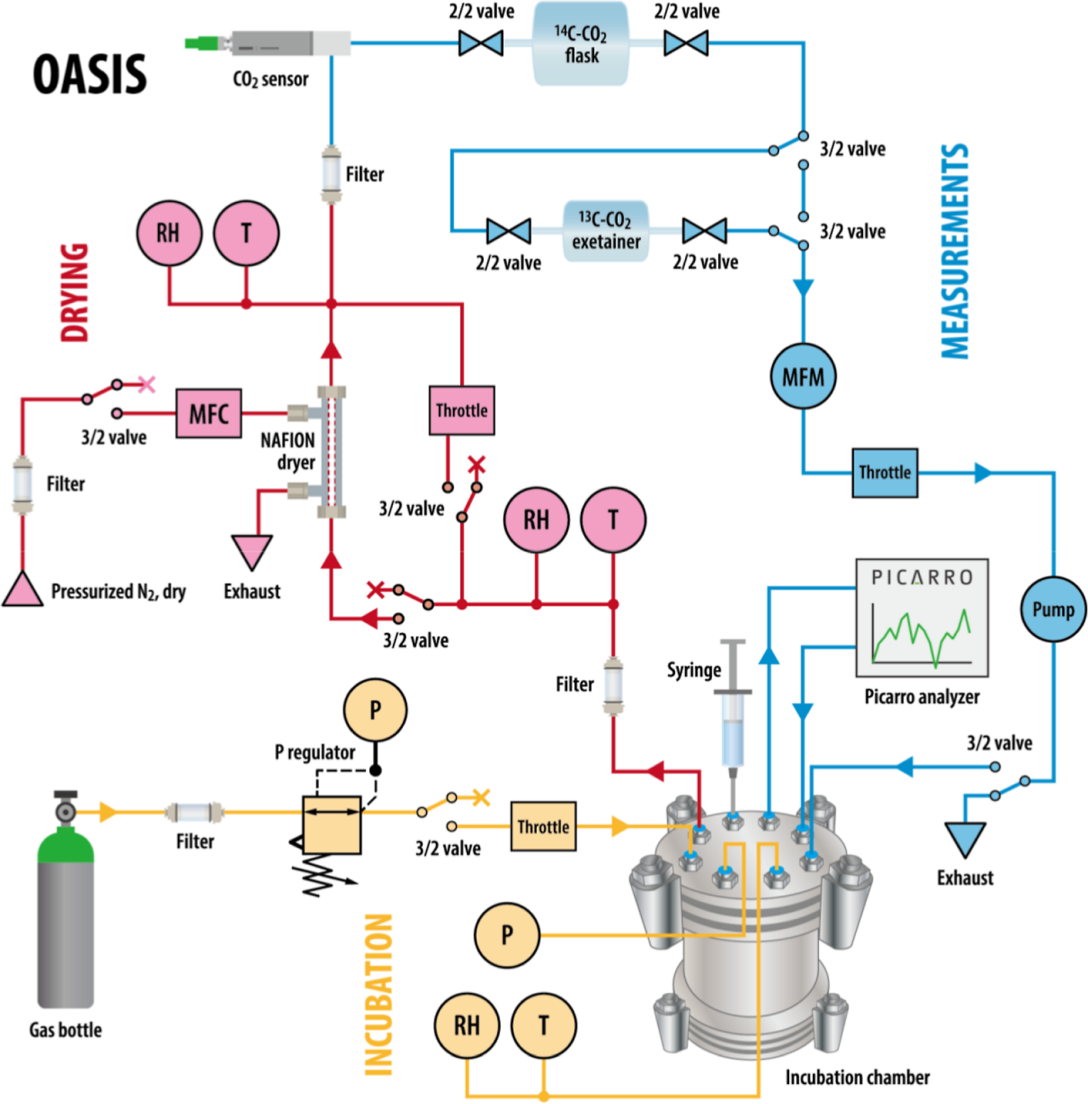
Schematic diagram of the Online Automatic Soil Incubation System (OASIS). It includes three modules: (1) the measurement module (blue) for real-time CO_2_ analysis and gas sampling for isotopic analysis; (2) the drying module (red) for manipulation of drying; (3) the Incubation section (yellow) for soil incubation and monitoring of environmental conditions. *P*, Pressure manometer; RH, Relative Humidity; T, Temperature; MFC, Mass Flow Controller; MFM, Mass Flow Meter.

### Soil Collection

The soil samples were collected from a mature forest stand established in 2002 at the Yangjuangou catchment on the Loess Plateau, Shanxi Province, China (36°42′ N, 109°31′ E). The study area experiences a semiarid continental monsoon climate, with an average annual temperature of 9.4 °C and an average annual precipitation of 527 mm. Most of the precipitation occurs between July and September, typically in the form of heavy storms characterized by a high intensity and short duration. In October 2022, we established four sampling plots (2 m × 2 m) beneath the canopy of the dominant tree species, Robinia pseudoacacia. Within each plot, after removing surface litter, we excavated a small pit to expose the soil profile and marked the 0–10 cm layer with a ruler. We then collected three random subsamples from this depth by sampling horizontally along the exposed face with a clean spade. The three subsamples were pooled and thoroughly mixed in the field to form one composite topsoil sample per plot (∼1 kg). In the laboratory, the soil samples were air-dried at room temperature (approximately 20 °C) and sieved to <2 mm, with visible dead roots and other debris carefully removed. Separately, at each plot, three intact soil cores were collected from the 0–10 cm layer using a core sampler (100 cm^3^ volume) to determine soil bulk density and water holding capacity (WHC). Subsamples for chemical and isotopic analysis were taken from each of the four sieved composite soils before the final mixing for incubation experiments and subsequently ground with a ball mill (Retsch MM400).

### Analysis of SOC, SIC, and Their ^13^C

The content of total soil carbon was determined by combustion at 1100 °C using a CN analyzer (Vario Max, Elementar Analysensysteme GmbH). The content of soil inorganic carbon (SIC) was measured using the same method after removing SOC at 450 °C for 16 h. The δ^13^C of total soil carbon was measured on bulk soil subsamples, whereas the δ^13^C of SOC was determined on samples treated with 1 M HCl to remove SIC. Both analyses were conducted using an isotope ratio mass spectrometer (IRMS; Delta + XL; Thermo Fisher Scientific).^23^ The soils used in this study contained 0.69% SOC and 1.46% SIC. The δ^13^C values of total carbon, SOC, and SIC were −10.31‰, −20.41‰, and −5.47‰, respectively.

### Drying-Rewetting Treatment and Incubation

Throughout this study, all soil moisture levels are expressed as gravimetric water content (GWC, %), calculated as the mass (g) of water per 100 g of dry soil. Manipulation of GWC was achieved via two drying-rewetting regimes: normal drying-rewetting cycles (NDWC) and extreme drying-rewetting cycles (EDWC). The NDWC treatment consisted of three sequential cycles, including one moderate drying-rewetting event (10.7% to 32% GWC) and two small drying-rewetting events: soils were dried to a target of 10.7% GWC and then rewetted to 15%. The EDWC treatment was characterized by a strong rewetting (2% to 32%) followed by a prolonged drying event over 7 days to a target of 2% GWC (Figure 2). Despite the different intensity, duration, and frequency of drying-rewetting, the total amount of water input was consistent for NDWC and EDWC. This allows our study to manipulate changes in precipitation patterns, i.e., the intensity, duration, and frequency of drying-rewetting, while controlling for total water input. This is because the frequency and intensity of precipitation and dry extremes have increased, while no significant trends have been observed in the total amount of precipitation.^24^ Both treatments were repeated for three consecutive cycles.

**Figure 2:**
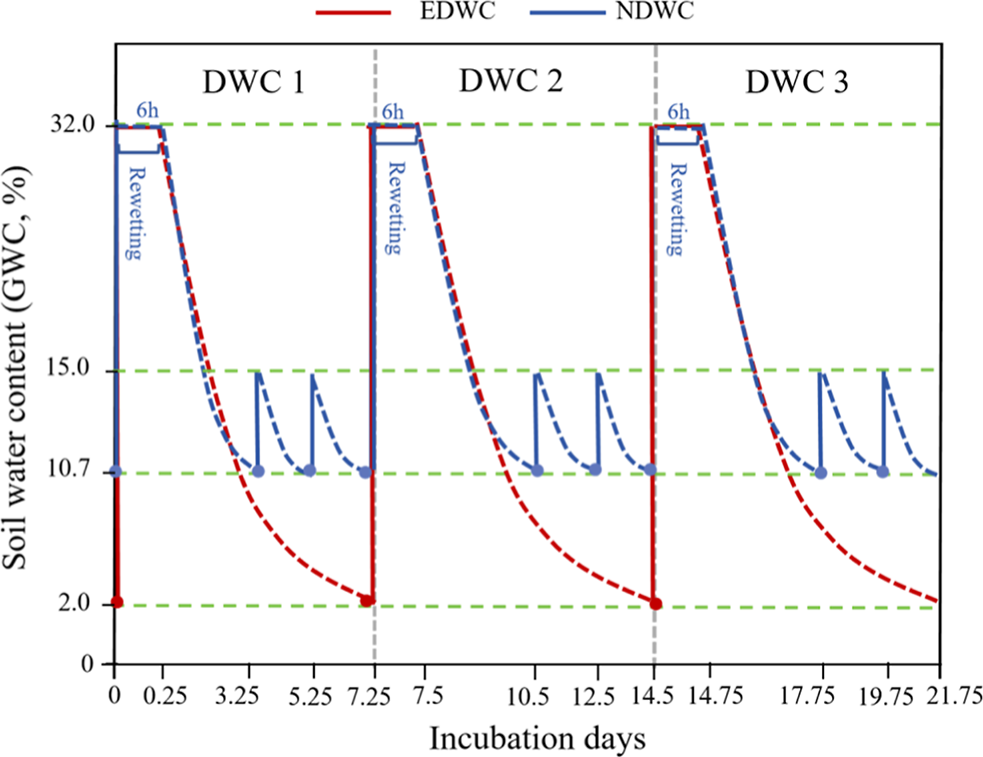
Schematic diagram of the experimental design for the two contrasting drying-rewetting cycle (DWC) scenarios. The blue and red lines represent the targeted soil water content (GWC, %) for the Normal (NDWC) and Extreme (EDWC) Dry-Rewetting Cycle treatments, respectively. For both treatments, dashed lines indicate the progressive drying phases, while solid vertical lines represent rewetting events. Each rewetting event is also marked by a circle.

Changes in GWC under the NDWC regime were designed to mimic fluctuations of GWC caused by normal rainfall and drying events in a typical monsoon season (e.g., September 2015, June–July 2016, and August–September 2018; Figure S2a), whereas changes in GWC under the EDWC regime were designed to mimic an extreme summer drought followed by a larger rewetting event (e.g., June–August 2015, August–September 2016, and June–July 2017; Figure S2a). The lowest soil water content threshold of ∼2% was frequently observed in the field. Regarding the upper threshold, although field measurements typically peaked at ∼20%, calculations based on the soil bulk density (1.12 g cm^–3^) indicate that the theoretical field capacity ranges from 30.9% to 36.1% (Figure S2a and Text S1). Consequently, the 32% target was selected to represent the realistic physical limit of water retention (field capacity) following an extreme rainfall. The treatments in this study were not intended to exactly replicate field conditions or quantify CO_2_ fluxes from the entire soil profile, but rather to create contrasting soil water conditions resulting from different intensities and durations of drying and rewetting to investigate the underlying source mechanisms.

The temperature was kept at ca*.* 20 °C throughout the incubation for both treatments. This temperature was chosen to represent the mean air temperature of the growing season (June to September) at the study site (see Figure S3 for detailed field temperature data). An aliquot (60 g) of dry soil was weighed into beakers and placed in each soil chamber. We used 60 g of soil per chamber to ensure collection of enough CO_2_ for Δ^14^C–CO_2_ measurements, while avoiding excessive CO_2_ build-up in the system.^25,26^ The linearity of CO_2_ emissions was confirmed by incubations with 30 and 60 g of soil samples (Figure S4). Water was added to the soils via a syringe (outside the chamber) connected to a custom-designed distributor inside the chamber. The distributor consisted of a rotatable Y-fitting with two outlets positioned at different heights. Water was injected slowly from the syringe while the Y-fitting was simultaneously rotated to achieve uniform distribution across the soil surface.

Before the start of each drying-rewetting cycle, soil chambers with nonsterilized soils were flushed with CO_2_-free synthetic air to avoid interferences on isotopic analysis. To minimize potential artifacts from the increased concentration gradient following CO_2_-free air flushing (conducted 6 h after rewetting), data from the first 4 h immediately following flushing were excluded from the kinetic analysis of CO_2_ release rates (Figure 5), but were accounted for cumulative emission calculations.

**Figure 3:**
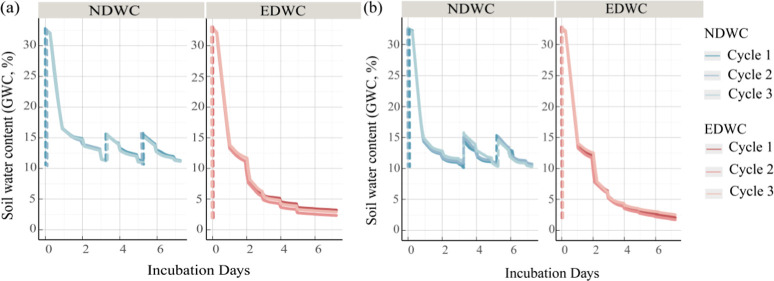
Dynamics of measured soil water content (GWC, %) during the incubation. The plots show the GWC in (a) sterilized soils and (b) nonsterilized soils under the two contrasting treatments. The blue lines represent the Normal Dry-Rewetting Cycle (NDWC) and the red lines represent the Extreme Dry-Rewetting Cycle (EDWC). The vertical dashed lines indicate the rewetting events.

**Figure 4:**
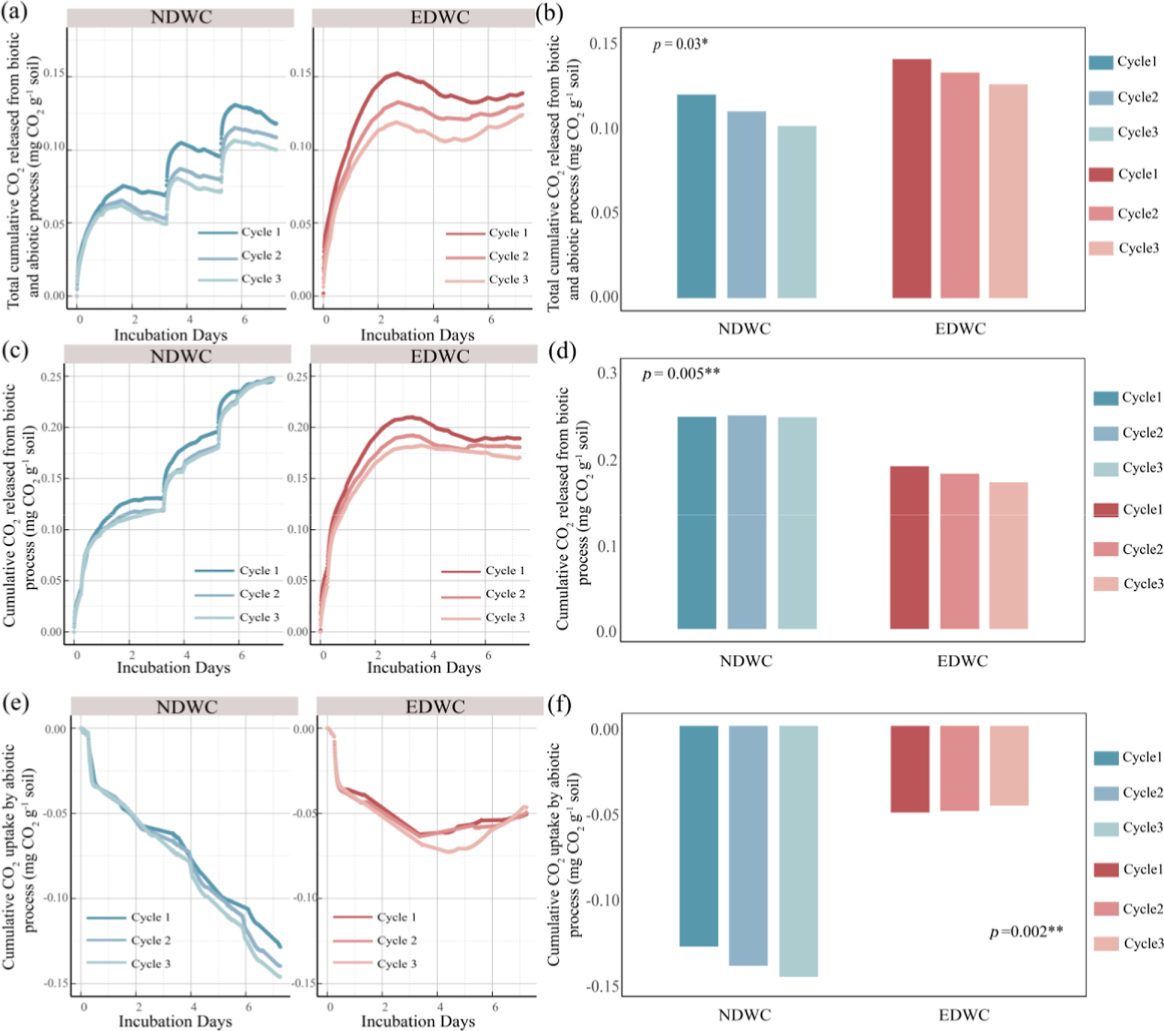
Magnitude of CO_2_ fluxes under normal drying-rewetting cycles (NDWC) and extreme drying-rewetting cycles (EDWC). (a,b) Total cumulative CO_2_ released from biotic and abiotic processes. (c,d) Cumulative CO_2_ released from biotic processes (CO_2_ released from nonsterilized soil minus CO_2_ uptake by sterilized soil). (e,f) Cumulative CO_2_ uptake by abiotic processes. Negative values indicate influx of CO_2_ into the sterilized soil. Different colored lines represent different cycles. Symbols *, **, and *** denote statistically significant differences between NDWC and EDWC treatments at the *p* < 0.05, *p* < 0.01, and *p* < 0.001 levels, respectively.

**Figure 5:**
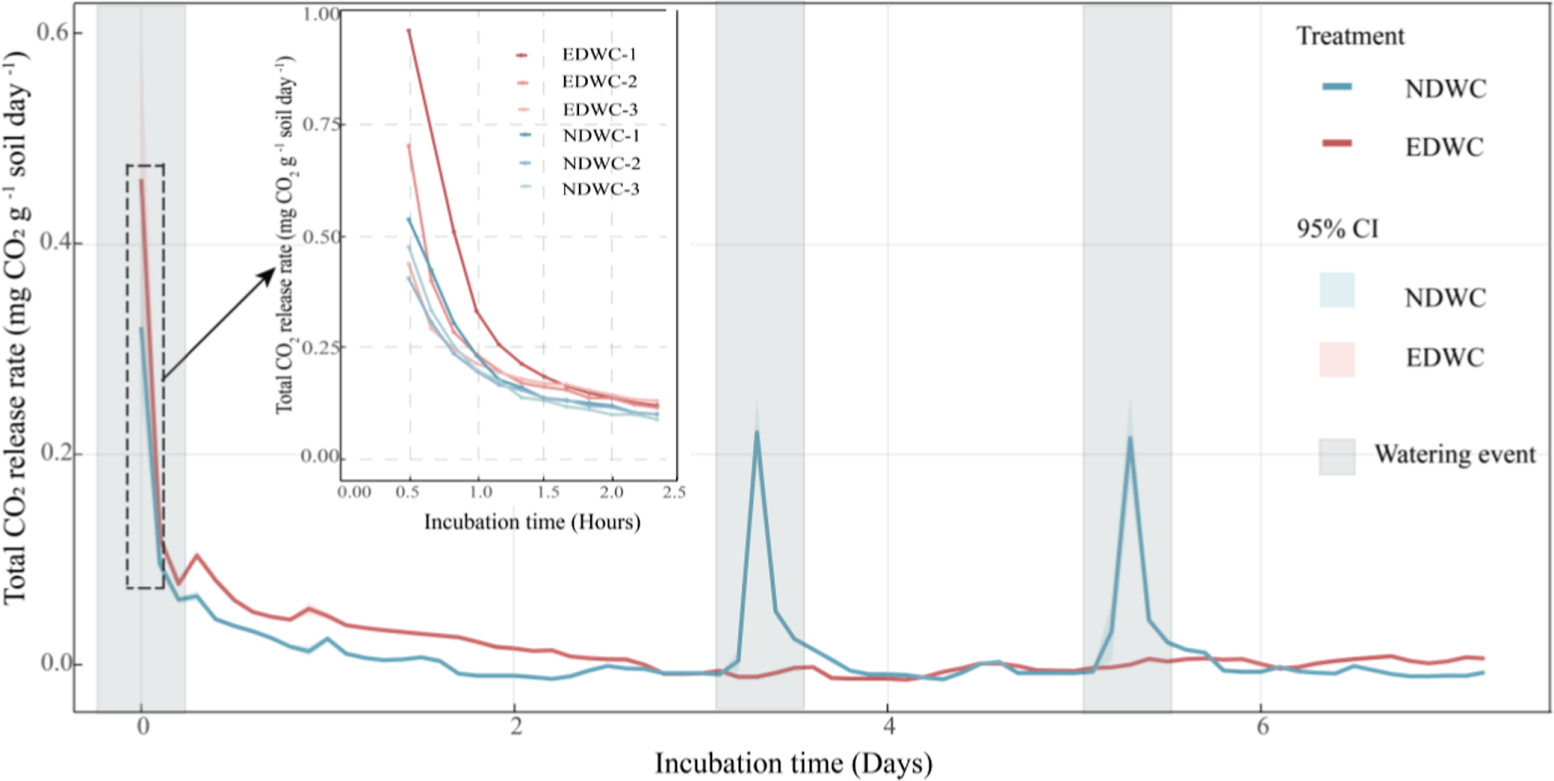
Total CO_2_ release rates (mg CO_2_ g^–1^ soil day^–1^) under NDWC and EDWC treatments during the incubation period. The subplot illustrates the magnitude of transient Birch effect responses following the first watering event in each of three experimental cycles for both treatments. The CO_2_ release rates were smoothed using the zoo:rollapply function, employing a moving average method with five data points.

To isolate the influence of biotic versus abiotic processes on CO_2_ fluxes, a parallel set of incubations was conducted using sterilized soils. An aliquot of dry soil (60 g) was placed in an autoclavable bag and sterilized via an autoclave (Autoclave DX45, Systec). The sterilization protocol consisted of heating to 121 °C at a pressure of 99.7 kPa for 60 min. This cycle was repeated three times prior to the experiment, with an interval of at least 24 h between each cycle. This intensive, repeated autoclaving protocol is a standard and highly effective method designed to maximally suppress biological activity by eliminating vegetative cells and resistant spores that germinate between sterilization cycles.^27^ Our analysis showed that sterilization had no significant effects on SOC and SIC content, while concentrations of DIC decreased from 123.6 to 80 mg/L (Table S1). Chambers with sterilized soil were flushed with a certified standard gas (synthetic air containing approximately 900 ppm of CO_2_) multiple times within each cycle whenever the CO_2_ concentration dropped below 400 ppm, to account for continuous CO_2_ uptake by soils. The range of CO_2_ in the sterilized soil chambers was chosen to match that observed in the nonsterilized chambers (400–900 ppm).

### Radiocarbon Analysis

The Δ^14^C of SOC and respired CO_2_ was measured using the accelerator mass spectrometry (AMS) facility at the Max Planck Institute for Biogeochemistry (MPI-BGC), Jena, Germany.^28^ For the ^14^C analysis of SOC, the bulk soil samples were weighed to yield ∼1 mg of organic carbon, based on their SOC content (0.69%). The procedure involved acid-washing (1 M HCl) to remove carbonates,^28^ followed by combustion. All gas samples were purified on a vacuum line to remove water vapor and trap CO_2_, and pure CO_2_ samples were graphitized via Fe/Zn reduction, and analyzed by MICADAS AMS (Ionplus AG, Switzerland) with Oxalic Acid II standardization and δ^13^C-based fractionation correction.^29^ The Δ^14^C values of SOC and SIC were −278.5‰ and −896.8‰, respectively. For the Δ^14^C analysis of CO_2_, gas samples were collected by detaching the 2.3 L sampling flask from the circulation system. The flask contained CO_2_ accumulated from both the drying phase and the subsequent rewetting phase (i.e., the first 6 h after rewetting). The ^14^CO_2_ sampling flask was not detached at the end of the drying phase or at the beginning of rewetting, because detaching the flask would have prevented precise, high-resolution δ^13^C measurements of CO_2_ (requiring CO_2_ >350 ppm) and thus compromised our ability to capture rapid changes in isotope sources immediately after rewetting. The gas samples were purified, graphitized, and analyzed for ^14^C following the same procedure as for soil samples. Analytical uncertainties for Δ^14^C were generally small, typically within ±5‰ for values in the range −100 to 0‰. ^14^C results follow standard nomenclature (F^14^C, Δ^14^C), with correction for mass-dependent isotope fractionation and normalization to internationally recognized standards; background was corrected using ^14^C-free organic and inorganic blanks.^28^ Radiocarbon data were reported as Δ^14^C in per mil (‰), defined as

1 Δ14C=(C14/12CsampleC14/12Coxalic−1)×1000

where the oxalic acid standard refers to the year 1950.^30^ To account for mass-dependent fractionation, all ^14^C/^12^C ratios were normalized to δ^13^C = −25‰.^26^

The fraction modern (*F* ^14^C) was calculated from Δ^14^C as

2 $$F^{14}C=\left(\frac{\Delta^{14}C}{1000}+1\right)\times e^{-\lambda \left(1950-Yc\right)}$$

where λ refers to the decay constant (1/8267 yr^–1^) and Yc refers to the collection year.

Conventional radiocarbon age (τ, years) was computed based on the radiocarbon decay

3 $$\tau =-8033\times \ln\left(F^{14}C\right)$$

where 8033 yr is derived from the Libby half-life 5568 yr.^31^

### ^13^C Data Processing and Analysis

For the ^13^C–CO_2_ analysis, the Picarro CRDS analyzer recorded high-frequency (ca*.* 1 Hz) raw data of the CO_2_ concentration and δ^13^C. Data were excluded when chamber CO_2_ concentrations were below 350 ppm. The filtered data were averaged into 10 min intervals. The δ^13^C signature of respired CO_2_ was calculated using a mass balance approach (eq 4), based on the differences in CO_2_ concentration and δ^13^C between two consecutive 10 min intervals. This method is inherently robust to small instrumental drift, as any measurement drift over hours or days is canceled out when calculating changes between consecutive 10 min intervals.

The δ^13^C signature of released CO_2_ was calculated by using the following mass balance

4 $$released{\;\delta }^{13}C=\frac{{\left[{CO}_{2}\right]}_{t}\times \delta^{13}C_{t}-{\left[{CO}_{2}\right]}_{t-\Delta t}\times \delta^{13}C_{t-\Delta t}}{{\left[{CO}_{2}\right]}_{t}-{\left[{CO}_{2}\right]}_{t-\Delta t}}$$

where [CO_2_]_*t*_ and [CO_2_]_*t*–Δ*t*_ represent the CO_2_ concentrations measured at two time points separated by a time interval of Δ*t*, while δ^13^C_*t*_ and δ^13^C_*t*–Δ*t*_ represent the δ^13^C–CO_2_ values measured by Picarro at the corresponding time points.

To estimate the fractions of CO_2_ derived from the SIC pool (F_SIC_) and SOC (F_SOC_), we applied a simple two-end-member isotopic mass-balance model. We assumed that the measured δ^13^C of CO_2_ (δ^13^C–CO_2_) in the headspace reflects mixing between CO_2_ derived from SIC (δ^13^C–SIC _derived_) and CO_2_ derived from SOC (δ^13^C–SOC), respectively.

5 $$\delta^{13}C-{CO}_{2}=\delta^{13}C-{SIC}_{derived}\times F_{SIC}+\delta^{13}C-SOC\times F_{SOC}$$

6 $$F_{SIC}+F_{SOC}=1$$

where δ^13^C–SIC _derived_ represents the isotopic signature of CO_2_ derived from SIC,^32^ calculated as

7 $$\delta^{13}C-{SIC}_{derived}=\delta^{13}C-SIC-9‰$$

### Soil CO_2_ Release Rate

Soil CO_2_ release rate (Rs, mg CO_2_ g^–1^ soil day^–1^) was calculated using the following equation

8 $$Rs=\frac{\Delta C\times V\times {MW}_{{CO}_{2}}\times 1000}{\Delta t\times M_{soil}\times Vm\times 10^{6}}$$

where Δ*C* is the change in CO_2_ concentrations (ppm), *V* is the total volume of the incubation system (3.35 L), MW_CO2_ is the molar mass of CO_2_ (44.01 g mol^–1^), Δ*t* is the time interval (days), *M*_soil_ is the dry mass of soil (60 g), and *V*_m_ is the molar volume of CO_2_ under experimental conditions (24.37 L mol^–1^), calculated using the ideal gas law

9 $$V_{m}=\frac{R\times T}{P}$$

where *R* is the gas constant (0.08314 L·bar mol^–1^ K^–1^), *T* is temperature (293.15 K), and *P* is pressure (1 bar).

### Statistical Analyses

The CO_2_ concentration and isotope data were averaged over the three drying-rewetting cycles to test for significant differences in these variables between the EDWC and NDWC treatments. Welch’s two-sample *t*-test and one-way analysis of variance (ANOVA) were used to compare the differences in cumulative soil CO_2_ emissions between the NDWC and EDWC treatments. Differences in Δ^14^C between the two treatments were assessed using an independent sample *t*-test. The relationships between changes in respiration (Δ*R*_s_) and soil moisture (ΔMoisture) were quantified through Bayesian linear mixed-effects modeling (brms package), with cycles included as random intercepts to account for repeated measurements. Posterior distributions were sampled via four MCMC chains (4000 iterations each). Group differences in Δ*R*_s_ across soil moisture intervals were evaluated via Kruskal–Wallis test with Dunn-Bonferroni posthoc adjustments, accompanied by epsilon-squared effect size calculations. All data analyses were conducted in R (version 4.3.0, R Core Team, 2023).

## Results and Discussion

### Simulation of Contrasting Drying-Rewetting Patterns

As expected, the OASIS system generated contrasting drying-rewetting regimes, while maintaining identical total water inputs across treatments (Figure 3). Overall, soils under EDWC treatment were exposed to more intensive rewetting and drying, as well as longer dry periods, compared to soils under NDWC. EDWC treatment induced rapid changes in soil moisture, where gravimetric water content (%, w/w) declined from 32.75% (±0.15%, *n* = 6) to 10.83% (±1.23%) within 2 days, followed by progressive drying to 2.45% (±0.53%) by the end of incubation. In contrast, NDWC treatment exhibited gradual changes in soil moisture: drying from 32.59% (±0.06%) to 11.1% (±0.58%) over 3 days, rewetting to 15.34% (±0.47%), subsequent drying to 10.80% (±0.31%) over the next 2 days, rewetting to 15.13% (±0.31%), and finally maintaining at 10.82% (±0.45%). Compared to the target levels, the OASIS system exhibited a small mean absolute error of 0.45% (±0.21%) across all drying-rewetting phases. Maximum deviations did not exceed +0.75% (EDWC initial phase) or +0.59% (NDWC first drying phase). Welch’s *t*-test confirmed no statistically significant differences between observed and target moisture levels (EDWC: *p* = 0.86; NDWC: *p* = 0.77; *n* = 6), indicating the high precision and reliability of the OASIS system in controlling the intensity and duration of soil drying and rewetting.

### Extreme Drying and Rewetting Alter the Magnitude and Temporal Patterns of CO_2_ Fluxes

Consistent with our hypothesis, the severe drying and greater increase in soil moisture under EDWC induced greater CO_2_ pulses during the first 2 days after rewetting, compared to NDWC (Figure 4a,c). It has been proposed that CO_2_ pulses upon rewetting are driven by microbial growth or by increased availability and accessibility of labile substrates, such as osmolytes accumulated during drying and compounds released during cell lysis, especially under EDWC.^7,8^ By continuously monitoring CO_2_ changes after rewetting, we found that CO_2_ release rates peaked immediately (within 10 min) after rewetting (Figure 5), in contrast to microbial biomass growth that may peak several hours later than respiration.^12,33,34^ This provides evidence supporting the accumulation of labile substrates during drying as the main process determining the magnitude of soil CO_2_ pulses after rewetting. Although diffusive transport, gas trapping, and oxygen limitation can influence CO_2_ emissions under field conditions, particularly in deep soils, they are unlikely to have affected our results. Rewetting to 32% GWC produced a water-filled pore space (WFPS) of ∼61% (based on a bulk density of 1.12 g cm^–3^ and a total porosity of 59%), leaving ∼23% air-filled porosity, which is sufficient for gas diffusion. Moreover, the linear increase in CO_2_ emissions from 30 to 60 g of soil (doubling soil depth) indicates that CO_2_ release in our study was not constrained by diffusive transport, gas trapping, or oxygen limitation (Figure S4). Notably, both the magnitude of CO_2_ pulse (Figure 4a,c) and release rates (Figure 5) decreased with increasing numbers of drying-rewetting cycles, especially under EDWC, suggesting progressive depletion of the substrates that accumulate during the drying phase.^8^

Compared to NDWC, the larger soil CO_2_ pulses following rewetting under EDWC were largely offset by lower CO_2_ emissions during the dry phase (Figure 4b), and even reversed when considering only CO_2_ emissions derived from biotic processes (Figure 4d). The total CO_2_ release remained relatively constant 3 days after rewetting under EDWC (Figure 4a), when soil moisture dropped below ca*.* 5% (Figure 3). This is likely because of suppressed microbial activity due to the reduced availability of water, carbon, and nutrients.^35,36^ While previous studies have often reported such offsets during the dry phase compared to controls with high and constant soil moisture,^11,37,38^ these studies cannot disentangle the effects of total water inputs from those of changing intensity and duration of drying-rewetting. By accounting for differences in water input, our study clearly shows that increased intensity and duration of rewetting and drying, which may occur under more intensive rainfall events and longer dry spells, can lead to stronger initial CO_2_ pulses, but these can be largely offset by reduced emissions during the dry phase. Nevertheless, EDWC led to a slight increase in cumulative CO_2_ emissions, indicating that, under the specific conditions used here, more extreme drying-rewetting may enhance overall CO_2_ emissions and soil carbon losses in the Loess Plateau. However, these results should be interpreted with caution, as the differences between EDWC and NDWC are sensitive to the prescribed duration and intensity of the wet and dry phases. For example, shortening the wet period or increasing the drying intensity in the EDWC treatment would likely reduce cumulative CO_2_ emissions to levels similar to or even lower than those under NDWC.

The CO_2_ release rates were strongly determined by the timing of rewetting (Figure 5) and the intensity of drying and rewetting (Figure 6). The severe drying and greater extent of soil rewetting under EDWC not only resulted in a higher peak in CO_2_ release within minutes after rewetting, but also sustained higher respiration rates during the first 2 days of the drying phase (Figure 5), despite a more rapid decline in soil moisture compared to NDWC (Figure 3). These results suggest that the intensity, duration, and timing of drying-rewetting events are more crucial than soil moisture alone in determining CO_2_ emissions from dryland soils. This starkly contrasts with the common empirical moisture-respiration functions used in Earth system models.^39,40^ Our results highlight the importance of high-resolution measurements of soil carbon flux and moisture content under varying drying-rewetting regimes to improve model representations of soil CO_2_ pulses and emissions in dryland ecosystems.^9,33,41^

**Figure 6:**
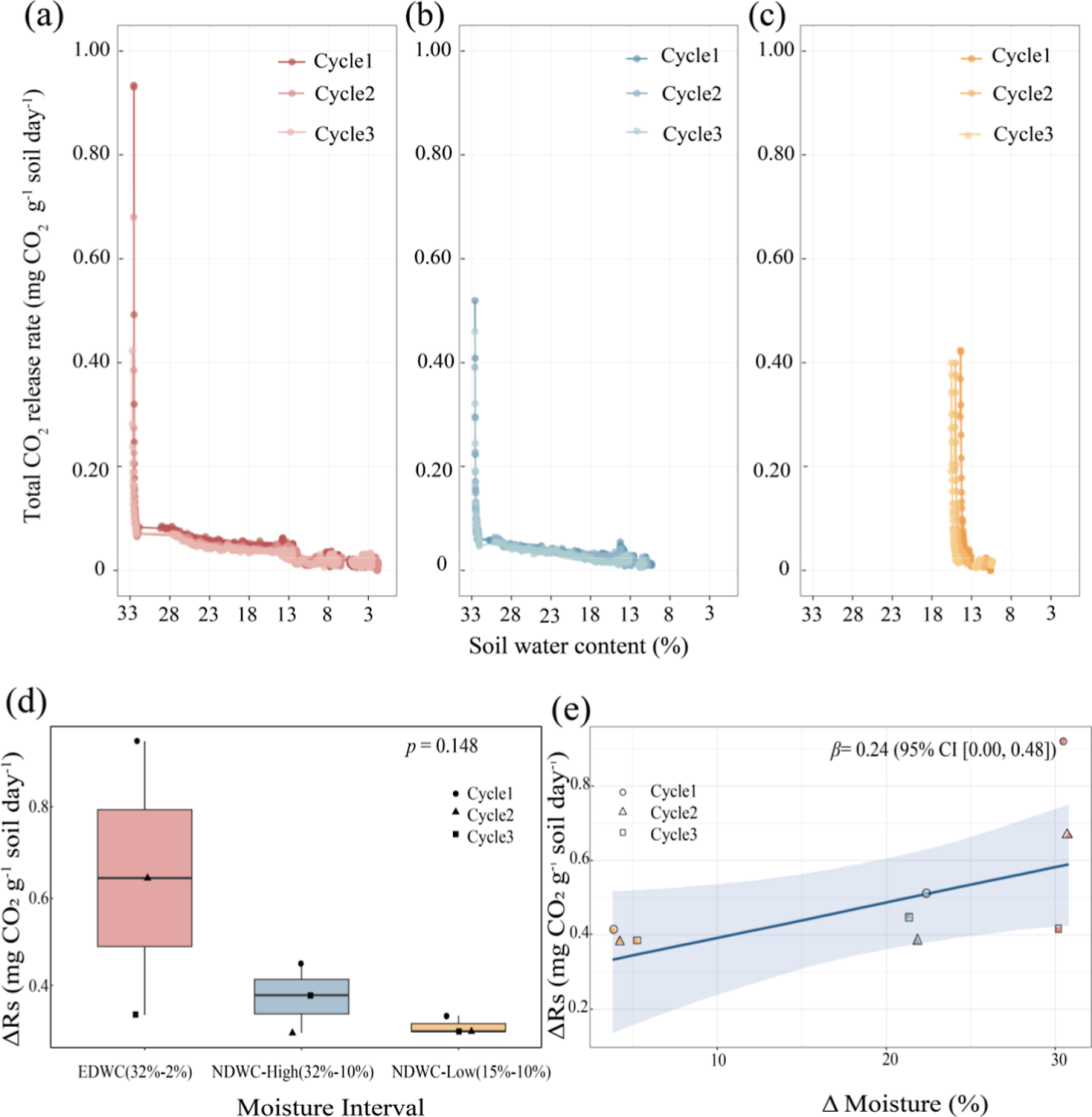
Response of total CO_2_ release rates and Birch effect dynamics to soil moisture gradients under different drying-rewetting cycle treatments. (a–c) CO_2_ release dynamics during soil moisture decrease. (a) Extreme drying-rewetting cycle (EDWC): Soil moisture decrease from 32% to 2% representing rewetting from severe drying (2%) to intensive rewetting (32%). (b) Normal drying-rewetting cycle (NDWC-High): Soil moisture decrease from 32% to 10.7%, representing rewetting from moderate drying (10.7%) to rewetting (32%). (c) Normal drying-rewetting cycle (NDWC-Low): Soil moisture decrease from 15% to 10.7%, representing rewetting from low drying (10.7%) to low rewetting (15%). (d) Magnitude of soil respiration change (Δ*R*_s_) across moisture intervals. Boxplots show median (central line), interquartile range (boxes), and individual measurements. Nonparametric analysis revealed marginal group differences (Kruskal–Wallis H = 3.82, *p* = 0.148; Dunn’s adjusted p for EDWC vs NDWC-Low = 0.158). (e) Bayesian conditional effects of moisture change (ΔMoisture) on Δ*R*_s_. Shaded band represents 95% credible interval of the posterior prediction (dark blue line: β = 0.24 mg CO_2_ g^–1^ soil day^–1^ per unit ΔMoisture increase). Points show raw data with interval-specific coloring. Model included Cycle as a random intercept (σ = 1.77, 95% CI [0.06, 5.92]), accounting for 18% of total variance (ICC = 0.18).

### Extreme Drying and Rewetting Reduce Sequestration of CO_2_ through Abiotic Processes

We observed significant CO_2_ influx in sterilized dry soils after rewetting (Figure 4e,f), which was of similar magnitude to the total CO_2_ efflux under NDWC. There is evidence that dryland soils can absorb CO_2_ from the atmosphere through an abiotic process like CO_2_ dissolution and carbonate formation process.^17^ In this process, CO_2_ reacts with H_2_O to form carbonic acid (H_2_CO_3_), dissociating into bicarbonate (HCO_3_^-^) and carbonate (CO_3_^2–^) ions, as well as hydrogen ions (H^+^). The bicarbonate and carbonate ions can react with alkaline minerals in the soil, such as calcium (Ca^2+^) and magnesium (Mg^2+^) ions, leading to precipitation of carbonates (e.g., CaCO_3_ and MgCO_3_). This process can be promoted by the relatively high pH (8.67) and large amounts of minerals (e.g., calcium) of the soils from the Loess Plateau. Significant uptake of CO_2_ by soils has also been observed in this region,^19^ which may contribute significantly to the total soil carbon pool.^42^ Notably, we did not observe net CO_2_ efflux from the sterilized soils under NDWC during the drying phase, indicating that CO_2_ dissolves and subsequently precipitates as carbonate with calcium mostly derived from noncarbonate sources (e.g., silicate weathering), rather than calcium carbonates whose dissolution and reprecipitation would result in zero net CO_2_ exchange.

The CO_2_ dissolution process may also explain why the magnitude of the CO_2_ influx was greater (i.e., more negative) in soils under NDWC treatment (−0.14 ± 0.05 mg of CO_2_ g^–1^ soil) than under EDWC treatment (−0.05 ± 0.01 mg of CO_2_ g^–1^ soil) (Figure 4f). However, there was a slight upward trend in the total CO_2_ flux during the dry phase under EDWC, indicating a small release of CO_2_ from the sterilized soils (Figure 4e). The upward trend in total CO_2_ flux during the dry phase under EDWC is possibly due to the shrinkage of water-filled pores and the drop in CO_2_ solubility during drying, which shift carbonate equilibrium and promote the release of CO_2_. This suggests that prolonged dry spells under climate change can limit abiotic CO_2_ uptake by soils and reduce CO_2_ sequestration in drylands like the Loess Plateau. Collectively, our results highlight the importance of accounting for the CO_2_ flux through abiotic processes, such as pedogenic carbonate formation, in understanding and modeling soil CO_2_ emissions and carbon sequestration in drylands.

### Isotopic Evidence Reveals a Shift in Carbon Sources of Soil CO_2_ Pulses during Drying-Rewetting

The mean Δ^14^C values of the respired CO_2_ were −52.20 ± 14.30‰ (corresponding to ca*.* 430 ± 120 years) and −43.30 ± 32.86‰ (ca*.* 360 ± 280 years) under EDWC and NDWC (Figure 7b), respectively, which are much higher than the Δ^14^C values of bulk SOC (corresponding to ca*.* 2600 years). This indicates that the CO_2_ emissions after rewetting and drying originated from a mixture of carbon fixed in the last decades (i.e., bomb-derived carbon since the 1960s) and carbon that has persisted for hundreds to thousands of years (bulk SOC or SIC).^13^ Our results contrast with previous incubation experiments reporting positive Δ^14^C values of respired CO_2_,^26^ which indicate major contributions from bomb-derived recent carbon since the 1960s. The old carbon in the released CO_2_ observed in our study is likely due to old SOC (Δ^14^C ≈ −280‰) and equilibration with old SIC (Δ^14^C ≈ −900‰). It should be noted that under natural conditions, the age of respired CO_2_ would likely be younger than observed here, given the fresh carbon inputs from plant litter and root exudates.^43^ Contrary to our hypothesis, the Δ^14^C of respired CO_2_ did not differ significantly between EDWC and NDWC, and exhibited large cycle-to-cycle variability that exceeded analytical uncertainty (around ±5‰), especially under NDWC. Although total CO_2_ emissions were likely dominated by biotic processes, i.e., microbial respiration of SOC, we speculate that even small differences in the contribution of abiotic processes, i.e. equilibration of headspace CO_2_ with strongly ^14^C-depleted SIC (Δ^14^C ≈ −900‰), could cause large differences in the Δ^14^C of CO_2_ accumulated over the entire cycle.

**Figure 7:**
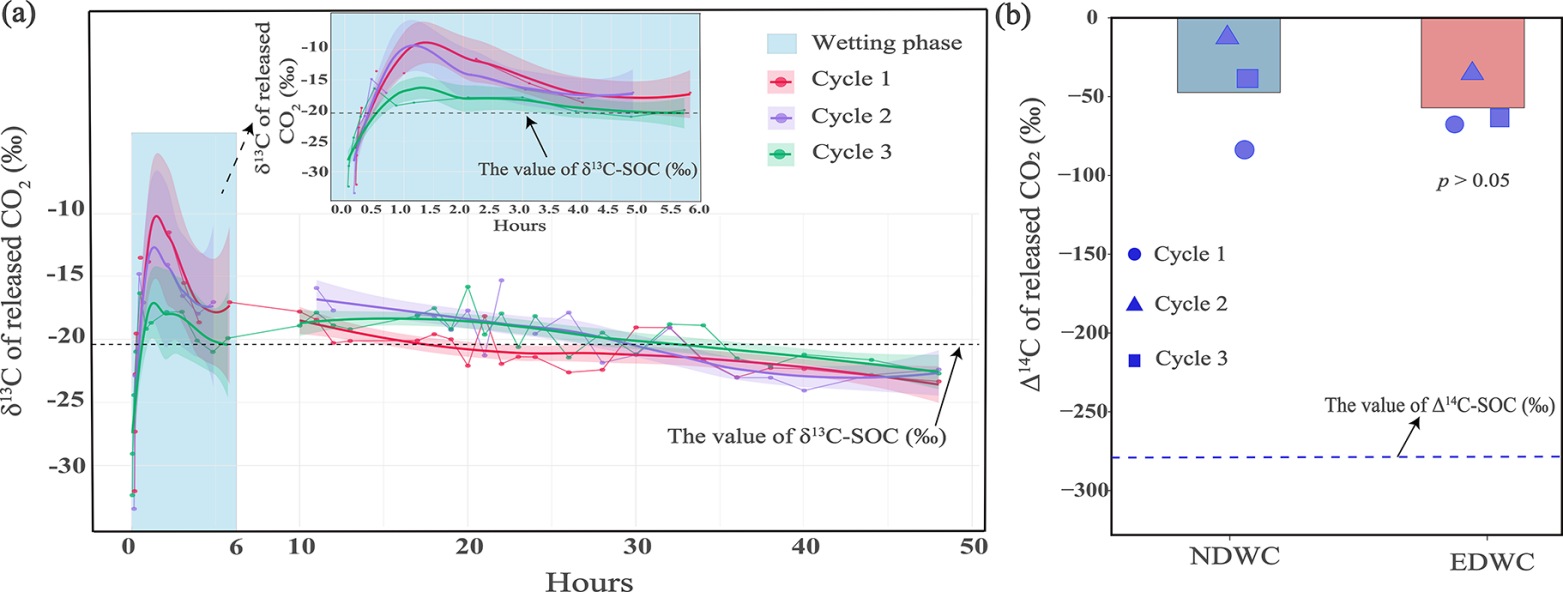
Isotopic signatures of cumulative CO_2_ emissions during incubation. (a) δ^13^C of released CO_2_ dynamics under Extreme drying-rewetting cycle (EDWC) treatment. Blue shaded areas denote the wetting phases. The inset panel provides an enlarged view of δ^13^C signatures during the initial 0–6 h wetting phase, revealing detailed temporal variations at higher resolutions. The black dashed line denotes the δ^13^C value of soil organic carbon (δ^13^C–SOC). (b) Δ^14^C–CO_2_ comparison between EDWC and Normal dry-rewetting cycle (NDWC) treatments. The blue dashed line represents the Δ^14^C value of bulk soil organic carbon (SOC). Data show measurements at the end of each dry-rewetting cycle.

The δ^13^C of respired CO_2_ under EDWC reflects shifts in the carbon substrates used for decomposition after rewetting. Within minutes of rewetting, the δ^13^C of respired CO_2_ was −32.60 ± 0.73‰ (Figure 7a). We speculate that the very negative δ^13^C signature observed within minutes of rewetting reflects the rapid reactivation of dormant microorganisms^44^ and their use of microbial osmolytes or soluble compounds released through cell lysis during drying.^12,45^ These compounds are expected to be more ^13^C-depleted than their plant-derived substrates, such as root exudates and fresh litter inputs (δ^13^C = −27.88‰). This may contribute to approximately 10% to 15% of the cumulative CO_2_ emissions. After half an hour of rewetting, the δ^13^C of the instantaneously released CO_2_ increased to approximately −16.8 ± 4.6‰ (Figure 7a), possibly reflecting kinetic fractionation and isotopic exchange of headspace CO_2_ with the ^13^C-enriched SIC pool. This interpretation is supported by an independent incubation experiment with sterilized soils, which showed comparable ^13^C enrichment of headspace CO_2_ due to exchange with the DIC pool (Figure S5). Nevertheless, closed-chamber experiments with isotopic labeling are needed to confirm the proposed kinetic fractionation and exchange with the SIC pool. This carbonate-related CO_2_ exchange appeared to be strongly suppressed during the third cycle. During the drying period (10 to 48 h), the δ^13^C of respired CO_2_ gradually declined from ca*.* −18.00 to ca*.* −22.00‰, indicating significant contributions from bulk SOM (−20.40‰). We note that under natural conditions, however, rainfall may induce the decomposition of plant litter and the release of root exudates, which increases the contributions of plant-derived labile carbon to the CO_2_ pulse. Our results highlight the importance of combining radiocarbon analysis and high-temporal-resolution measurements of stable isotopes to identify the multiple carbon sources contributing to the CO_2_ pulse under drying-rewetting.

## Environmental Implications

Here we introduce an online automatic soil incubation system (OASIS) and demonstrate its application in manipulating the intensity and duration of drying-rewetting, quantifying soil CO_2_ fluxes, and examining changes in carbon sources. We show that extreme drying-rewetting caused faster and larger initial CO_2_ pulse emissions, which were partly counterbalanced by low CO_2_ emissions from biotic processes during the prolonged dry period. This suggests that, as seasonal and annual rainfall variability increases,^24^ drylands are likely to become more dominant contributors to variability in terrestrial carbon fluxes. Earth system models (ESMs) should be improved to capture such rapid and intense CO_2_ fluxes, rather than relying on smooth soil-moisture response functions for respiration.^5^ Furthermore, our results suggest that there is substantial CO_2_ influx, possibly through pedogenic carbonate formation, but this abiotic CO_2_ sink may be limited under more extreme drying-rewetting regimes and depends critically on the source of new calcium (decomposition of plant matter, dust, and silicate weathering) that could form pedogenic carbonates. ESMs that focus primarily on biological processes and do not account for pedogenic carbonate formation may underestimate the carbon sink capacity of drylands or misattribute the mechanisms behind observed total CO_2_ fluxes.

Stable isotope data indicate that plant-derived, ^13^C-depleted carbon dominates the rapid CO_2_ release within minutes of rewetting, followed by kinetic fractionation and exchange during equilibration of CO_2_ with ^13^C-enriched SIC and DIC and, later, from SOM decomposition that dominates the evolution of ^13^CO_2_ during the drying phase. Radiocarbon (^14^C) data also indicate that respired CO_2_ is younger than bulk SOC and combines carbon fixed in recent decades with contributions from carbon that has persisted in soils for hundreds to thousands of years. Equilibration with very old SIC is slow and accounts for a small fraction of the CO_2_, but it could be reflected in the variability in ^14^CO_2_ measured. Overall, our results suggest that the CO_2_ pulse emissions observed after rainfall results from not only decomposition of recent plant carbon sources but also exchange with SIC and decomposition of SOM.

Although our study provides new insights into how long dry spells and intensive rainfall can influence the patterns, magnitudes, and sources of soil CO_2_ flux, it has several limitations. First, we acknowledge that using a single incubation temperature does not capture the full range of field temperature variability. Second, our experiments were conducted on topsoils from the Loess Plateau, so the patterns we observe may not directly apply to deeper layers or to soils with different texture, mineral composition, or carbonate contents. Third, we excluded plant inputs, such as litter and root exudates, which would likely enhance CO_2_ pulse emissions following rewetting and increase the contribution of recent carbon. To address these limitations, future work should investigate how interacting climatic (e.g., precipitation, temperature and N deposition), biological (e.g., litter and root exudates), and physicochemical factors (e.g., pH, soil texture) influence the fluxes and isotopic sources of greenhouse gases, including CO_2_, methane (CH_4_),^46^ and nitrous oxide (N_2_O).^47^ Furthermore, combining the OASIS features with measurements of soil physicochemical properties and microbial characteristics (e.g., osmolytes and microbial biomass) at high temporal resolution will provide new insights into how these interacting factors influence soil CO_2_ pulses and emissions, which are crucial for developing and validating mechanistic models.^12,33^

## Supplementary Material



## Data Availability

The data underlying this study are openly available in Zenodo at 10.5281/zenodo.16744015

## Notes

Additional experimental details, materials, and methods, including photographs of experimental platform setup (OASIS) (PDF)
