# Supplementary material for: Tracing Soil CO2 Fluxes under Drying-Rewetting Cycles: Isotopic Insights from an Automatic Soil Incubation System
Source: Environ Sci Technol. 2026 Mar 6;60(11):8482–93. doi: 10.1021/acs.est.5c10776 (PMC13019666; doi:10.1021/acs.est.5c10776)
Supplement: Supplementary file 1 [file es5c10776_si_001.pdf]

SUPPORTING INFORMATION

Tracing Soil CO_2 Fluxes under Drying-Rewetting Cycles: Isotopic Insights from an Automatic Soil Incubation System

Yuedan Zhao [a,b,c], Nan Lu [b,c*], Susan Trumbore [a], Martin Goebel [a], Karl Kuebler [a], Hui Wang [a], Marion Schrumpf [a], Kai Wang [b,c], Cong Wang [b,c], Bojie Fu [b,c], and Jianbei Huang [a*]

[a] Max Planck Institute for Biogeochemistry, Hans-Knöll-Straße 10, 07745 Jena, Germany

[b] State Key Laboratory of Regional and Urban Ecology, Research Center for Eco-Environmental Sciences, Chinese Academy of Sciences, Beijing 100085, China

[c] University of Chinese Academy of Sciences, Beijing 100049, China

* Corresponding authors: Nan Lu and Jianbei Huang

Email: nanlv@rcees.ac.cn; hjianbei@bgc-jena.mpg.de

This PDF file includes:

Number of pages: 9

Number of figures: 6

Number of tables: 1

Number of texts: 1

Figure S1. Photograph of the operational Online Automatic Soil Incubation System (OASIS) platform during experimental runtime. Key components include: (i) Soil incubation chambers with real-time environmental sensors, (ii) Nafion membrane drying module (semi-permeable membrane for water removal), (iii) Continuous CO_2 concentration analyzers, and (iv) Automated sampling unit for $\delta^{13}C$ and $\Delta^{14}C$ analysis. The integrated system achieves precise drying-rewetting control and high-frequency gas flux monitoring, with the schematic workflow detailed in Figure 1.

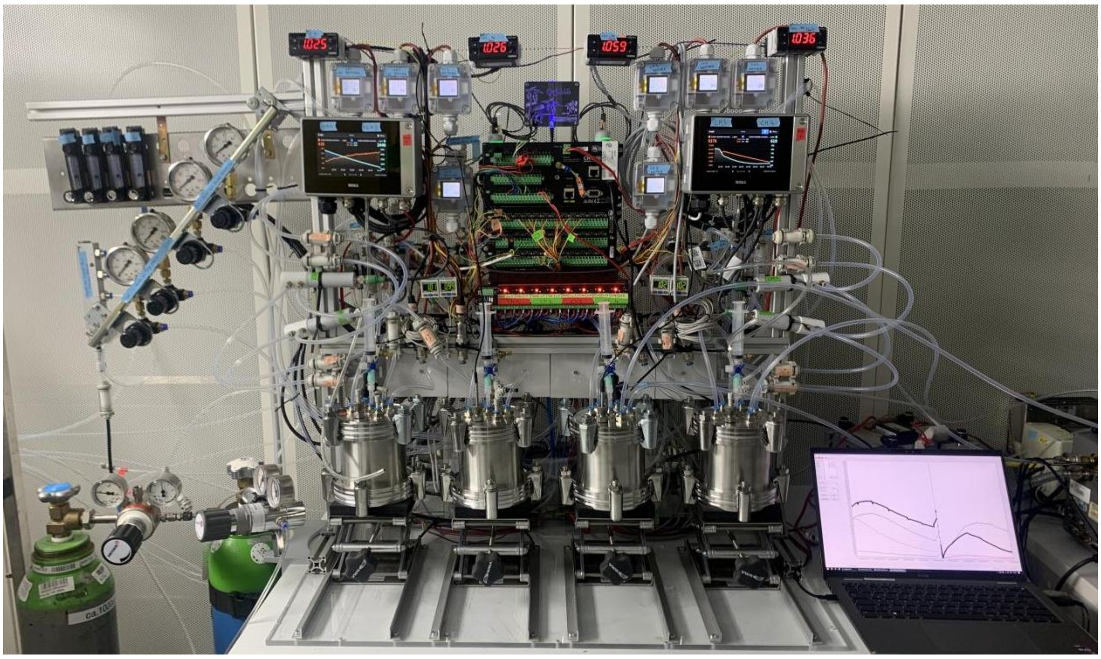

Figure S2. Statistical justification of the experimental drying-rewetting regimes based on long-term field observations (2014–2018). (a) Time series of topsoil (0–10 cm) gravimetric water content (GWC, %) during the growing season (June–September). Dashed horizontal lines indicate the target moisture levels used in the incubation: 2% (Extreme drying target, red), 10.7% (Normal drying target, orange), 15% (Normal rewetting target, blue), and 32% (Extreme rewetting target, green). The upper light-green shaded band represents the theoretical Field Capacity (30.9%–36.1%) derived from soil bulk density (1.12 g cm^{-3}), confirming that the 32% rewetting target represents the realistic upper limit of water retention following an extreme rainfall event. The yellow rectangular boxes indicate periods resembling the EDWC regime, and the green rectangular boxes represent periods resembling the NDWC regime. (b) Probability Density: The frequency distribution of field soil moisture confirms that the NDWC regime simulates the statistically most frequent (dominant) moisture state of the ecosystem. (c) Duration-Intensity Interaction: Analysis of discrete natural drying events confirms that drought intensity is conditional on duration. NDWC (Blue, Left): Short drying intervals (≤ 3 days) predominantly maintain the soil in a mesic state. In 68% of these events (n = 22), soil moisture remained above 10%, validating the NDWC target (10.7%) (blue solid line) as a representative baseline for frequent rainfall periods. EDWC (Red, Right): Prolonged intervals (≥7 days) are a necessary precondition to drive the soil into xeric conditions. In this regime (n = 28), moisture levels frequently drop below 5% (43% of events). The EDWC target (2%) (red solid line) aligns with the lower boundary (maximum intensity) of natural variability, confirming that a 7-day duration is required to simulate the physiological stress of extreme drought.

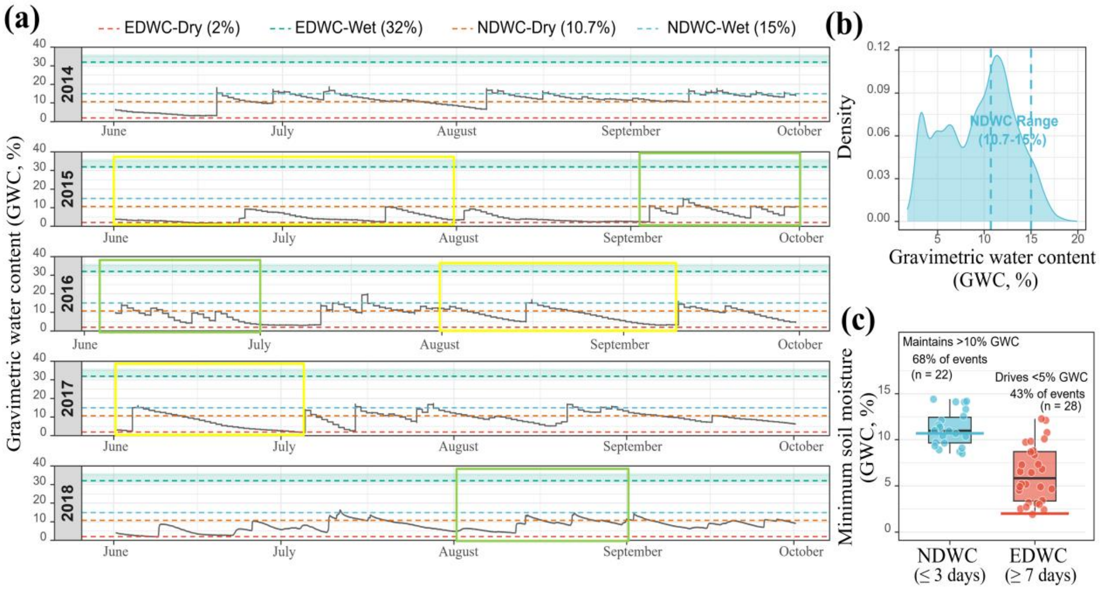

Figure S3. Field air temperature at the Yangjuangou catchment study site during the growing season (June–September) of 2021 and 2023. Boxplots visualize the distribution of daily mean temperatures, with overlaid colored points representing individual daily measurements (n denotes sample size). The solid horizontal line within each box represents the mean temperature. The horizontal dashed line indicates the constant 20 °C temperature used for the laboratory incubation, demonstrating its representativeness of typical growing season conditions at the field site. Data were recorded by an on-site automatic weather station.

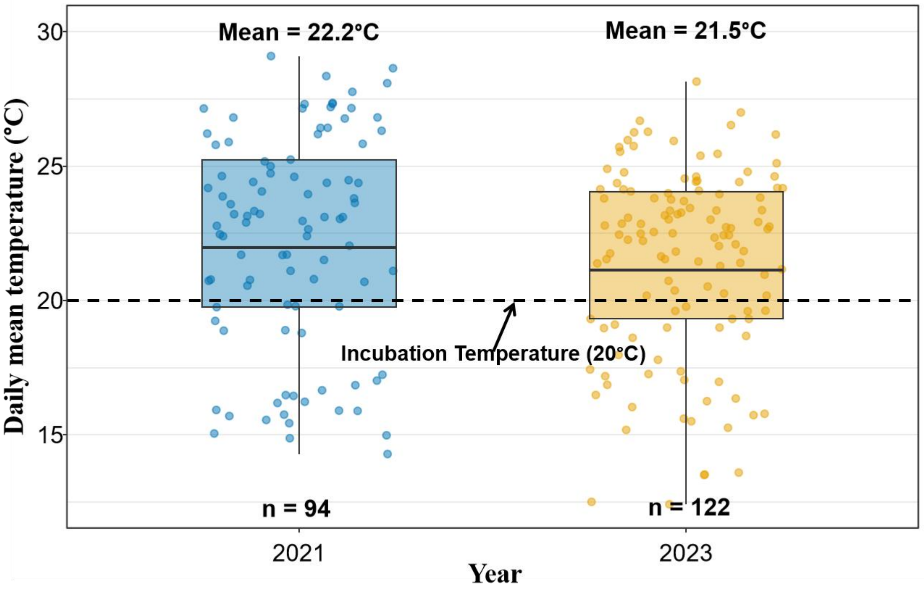

Figure S4. Comparison of cumulative CO_2 emissions from 30 g and 60 g soil samples following rewetting. Note that these soils come from the same region as the main experiment, but were collected from plots dominated by a different plant species (*Spiraea salicifolia*). Data points represent 10-minute aggregated averages of continuous measurements to visualize the steady-state emission trends.

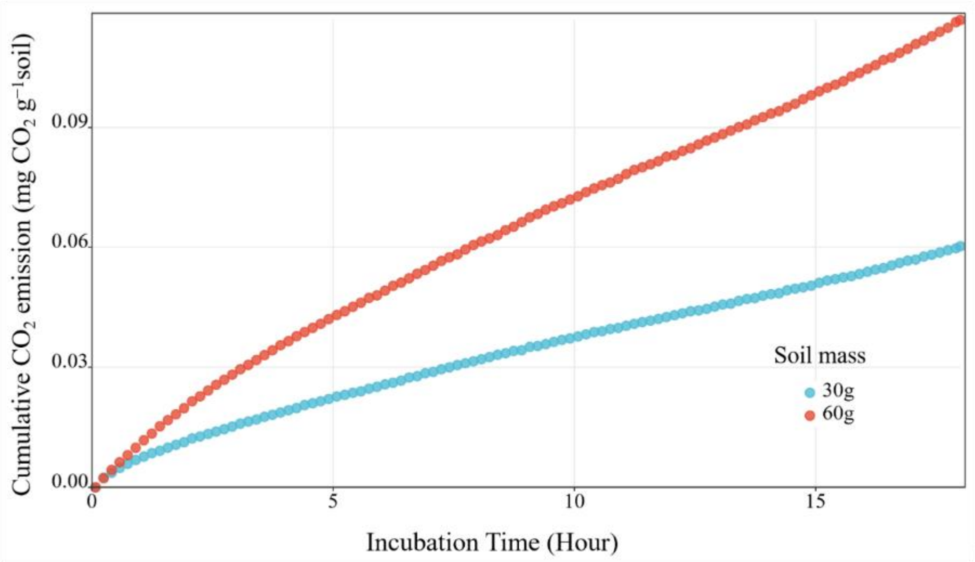

Figure S5. Additional experiment demonstrating rapid exchange of carbonate-derived carbon with headspace CO_2. Approximately 15 g of air-dried soil was placed in 1.08 L glass jars, which were sealed and flushed for 10 min (via a syringe needle through the septum) with CO_2 at 880 ppm and $\delta^{13}C = -36‰$. Afterwards, 5 g of deionized water was injected into the soil through a syringe needle. Jars were incubated at 20 °C in the dark, with three replicates of non-sterilized soil, two replicates of sterilized soil, and one blank jar without soil. Gas samples for CO_2 concentration and $\delta^{13}C$ were collected from the headspace using a syringe needle before water addition and approximately 15, 30, 60, 120, 240, and 480 min after wetting, and injected into pre-evacuated 12 mL Exetainers for subsequent isotopic analysis. In non-sterilized soils, $\delta^{13}C$ of CO_2 increased most strongly and CO_2 concentrations rose sharply, due to the combined effects of respired CO_2 and isotopic exchange between headspace CO_2 and soil inorganic carbon (SIC). In sterilized soils, however, CO_2 concentrations decreased slightly but $\delta^{13}C$ of CO_2 still increased, indicating rapid isotopic exchange between headspace CO_2 and SIC. Given that the initial $\delta^{13}C$ of headspace CO_2 in the sterilized treatment was −34.82‰ and increased to −32.49‰ after 30 min ($\Delta\delta^{13}C = 2.33‰$), and assuming $\delta^{13}C$ of CO_2 derived from SIC is −15‰ (see equations above), a simple two-end-member isotopic mass balance indicates that ~12% of the headspace CO_2 (~104 ppm of the initial 880 ppm) had exchanged carbon with SIC in 15 g of soil within 30 min, equivalent to ~ 0.65 mg CO_2 g^{-1} day^{-1}. This corroborates our results in the main experiment that CO_2 emissions observed in the first 30 min after rewetting (0.4–1 mg CO_2 g^{-1} soil day^{-1}) likely underwent substantial exchange with SIC.

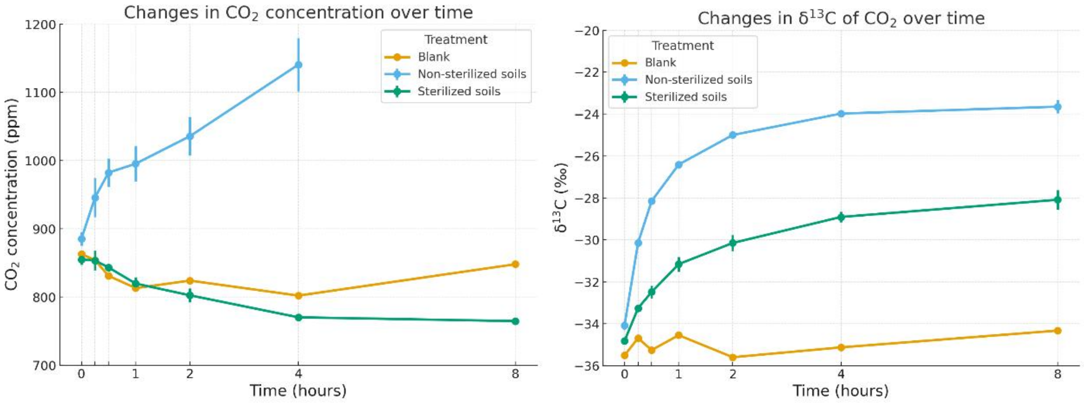

Figure S6. Model diagnostics for the Bayesian mixed-effects analysis. (a) Markov chain Monte Carlo (MCMC) trace plots of key parameters. Four chains (colored lines) demonstrate stable mixing for the ΔMoisture fixed effect (β) and residual standard deviation (σ). Convergence validated by Rhat = 1.00 and ESS >2,600. (b) Posterior predictive check of model fit. Observed ΔRs distribution (dark blue) aligns with 50 simulated trajectories (gray), supporting model adequacy (WAIC = 52.3, LOOIC = 52.1).

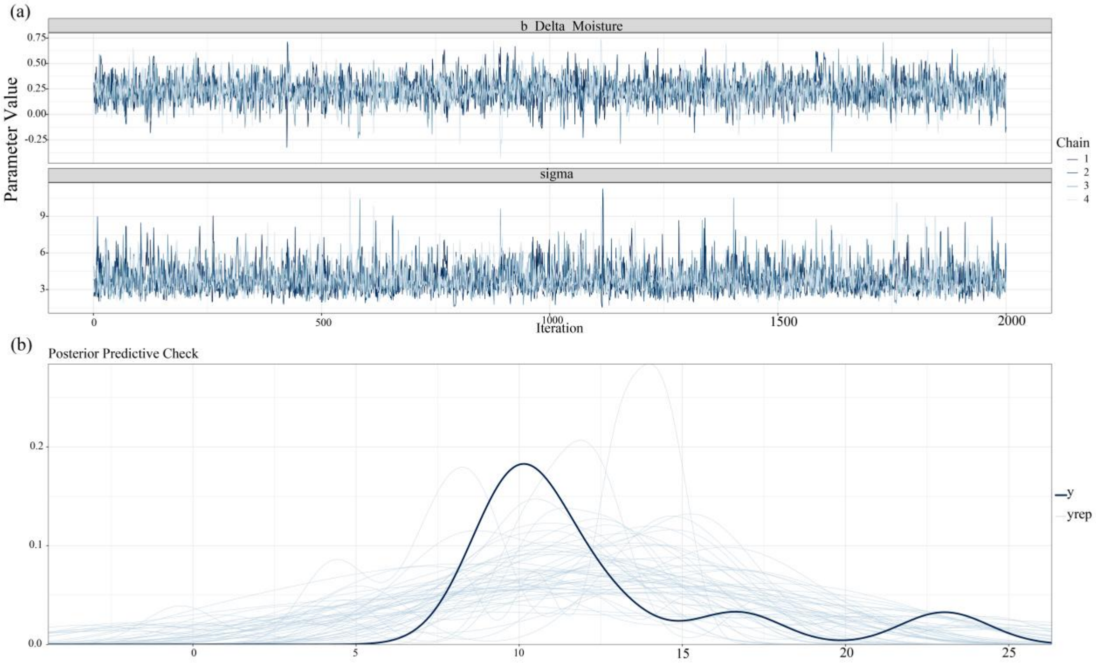

Table S1. The total soil carbon, inorganic carbon, dissolved inorganic carbon (DIC) and pH of non-sterilized and sterilized soils. Values are presented as mean ± standard error (SE, n = 3). The non-sterilized soils represent the biotic control, while sterilized soils were subjected to repeated autoclaving to inhibit biological activity. Soil pH values are reported based on water extraction (1:2.5 soil: water ratio).

	Total carbon (%)		Inorganic carbon (%)		Dissolved inorganic carbon (mg L^{-1})		pH	
	Mean	Standard Error	Mean	Standard Error	Mean	Standard Error	Mean	Standard Error
Non-sterilized soil	2.16	0.01	1.47	0.00	123.61	12.05	8.67	0.01
Sterilized soil	2.16	0.01	1.45	0.00	80.04	4.67	8.73	0.01

Text S1. Theoretical Derivation of Field Capacity.

To simulate the maximum water retention capacity (i.e., field capacity) following an extreme rainfall event, the experimental rewetting target was set at 32% gravimetric water content (GWC). This value was derived using standard soil physics protocols[1] through a stepwise calculation of soil porosity and saturation limits.

First, the total soil porosity (ϕ), representing the maximum volume fraction available for air or water, was calculated based on the relationship between bulk density (ρ_b) and particle density (ρ_p):

$$\phi = 1 - \left(\frac{\rho_b}{\rho_p}\right) \quad (1)$$

Here, ρ_b corresponds to the specific bulk density measured from intact soil cores at the study site (1.12 g cm^{-3}), and ρ_p is the standard particle density for mineral soils (2.65 g cm^{-3}), reflecting the dominance of quartz and aluminosilicates in the Loess Plateau mineralogy [1, 2]. Substituting these values yields a total porosity of 0.577 (or 57.7% by volume).

To determine the gravimetric water content at saturation (θ_s), which occurs when all soil pores are water-filled, we converted the volumetric porosity to a mass basis using the density of water ($\rho_w \approx$ 1.0 g cm^{-3}):

$$\theta_s = \frac{\phi}{\rho_b} \times \rho_w \quad (2)$$

This calculation yields a saturation GWC of 0.515 (51.5%), representing the theoretical maximum water holding capacity under ponded conditions.

Finally, the field capacity (FC), defined as the water content remaining after gravitational drainage (matric potential ≈ -33 kPa), was estimated based on the soil texture. Since the soils in the Loess Plateau are classified as silt loam, established hydraulic pedotransfer functions suggest that capillary forces retain water at levels corresponding to approximately 60%–70% of the saturation water content[3]. Applying this ratio to the calculated saturation value (51.5%) yields a theoretical field capacity range of 30.9% (51.5%×0.60) to 36.1% (51.5%×0.70). Consequently, our EDWC rewetting target of 32% falls squarely within this calculated range, confirming that the treatment realistically simulates the maximum physiological water availability following an intense storm while maintaining aerobic conditions.

References for Text S1:

(1) Hillel, D. Environmental Soil Physics; Academic Press, **1998**.

(2) Blake, G. R.; Hartge, K. H. Bulk Density. In Methods of Soil Analysis: Part 1—Physical and Mineralogical Methods; Klute, A., Ed.; SSSA Book Series; Soil Science Society of America, American Society of Agronomy, **1986**; pp 363–375.

(3) Saxton, K. E.; Rawls, W. J. Soil Water Characteristic Estimates by Texture and Organic Matter for Hydrologic Solutions. Soil Sci. Soc. Am. J. **2006**, *70* (5), 1569–1578.
